# Bulks of Al-B-C obtained by reactively spark plasma sintering and impact properties by Split Hopkinson Pressure Bar

**DOI:** 10.1038/s41598-019-55888-z

**Published:** 2019-12-20

**Authors:** O. Vasylkiv, H. Borodianska, D. Demirskyi, P. Li, T. S. Suzuki, M. A. Grigoroscuta, I. Pasuk, A. Kuncser, P. Badica

**Affiliations:** 1 National Institute for Materials Science, 1-2-1 Sengen, Tsukuba, Ibaraki 305-0047 Japan; 2 WPI-Advanced Institute for Materials Research (WPI-AIMR), Tohoku University, 2-1-1 Katahira, Aoba-ku, Sendai 980-8577 Japan; 3 James Watt School of Engineering, University of Glasgow, Glasgow, G12 8QQ UK; 4 School of Mechanical and Aerospace Engineering, Nanyang Technological University, Singapore, Singapore; 5 National Institute of Materials Physics, street Atomistilor 405A, 077125 Magurele, Ilfov Romania

**Keywords:** Composites, Mechanical properties

## Abstract

Mixtures of B_4_C, α-AlB_12_ and B powders were reactively spark plasma sintered at 1800 °C. Crystalline and amorphous boron powders were used. Samples were tested for their impact behavior by the Split Hopkinson Pressure Bar method. When the ratio *R* = B_4_C/α-AlB_12_ ≥ 1.3 for a constant B-amount, the major phase in the samples was the orthorhombic AlB_24_C_4_, and when *R* < 1 the amount of AlB_24_C_4_ significantly decreased. Predictions that AlB_24_C_4_ has the best mechanical impact properties since it is the most compact and close to the ideal cubic packing among the Al-B-C phases containing B_12_-type icosahedra were partially confirmed. Namely, the highest values of the Vickers hardness (32.4 GPa), dynamic strength (1323 MPa), strain and toughness were determined for the samples with *R* = 1.3, i.e., for the samples with a high amount of AlB_24_C_4_. However, the existence of a maximum, detectable especially in the dynamic strength vs. *R*, indicated the additional influence of the phases and the composite’s microstructure in the samples. The type of boron does not influence the dependencies of the indicated mechanical parameters with *R*, but the curves are shifted to slightly higher values for the samples in which amorphous boron was used.

## Introduction

Compounds, such as borides, with a strongly covalent character show excellent wear resistance, hardness, refractoriness, and chemical inertness properties. Boron carbide (BC) which is traditionally described by the chemical formula B_4_C fits this category. Boron carbide is also a light-weight material with the low density of 2.51 g/cm^3^. Although the fabrication of BC does not need high processing pressures as in the case of diamond or cubic BN, due to its covalent character, its processing temperatures are high and it is difficult to obtain high density parts with complex shapes. Under a compressive high velocity impact, the resistance of BC is considered to decrease due to the local process of amorphization^1^. The presented advantages and problems of BC, on the one hand, led to fabrication and assessment of new BC-based materials, and on the other hand, prompted the search for new materials with crystal structures inspired by BC. In the first case, strategies have been designed to overcome the difficulties by using additives for chemical substitutions in the crystal structure of boron carbide or for the formation of novel BC-based composites, in which the effort is directed towards control of the interfaces between the component phases. In the second case, of much interest are materials in the Al-B-C system^2–7^ especially those in which, similar to BC, there are icosahedral units. Icosahedral units are considered to play an important role in defining the outstanding mechanical properties of these materials. The most studied is the α-AlB_12_ phase composed of B_12_ icosahedra. This material can serve as a model for other structurally alike phases, e.g., AlB_24_C_4_ (known also in early refs. as AlB_10_). The crystal structure of the BC phases, namely α-AlB_12_ (Al_0.083_B), AlB_12_C_2_ (Al_0.5_B_6_C or Al_0.083_BC_0.167_) or AlB_24_C_4_ (Al_0.25_B_6_C or Al_0.0416_BC_0.167_) can be viewed as a stack of B_12_-like icosahedra packed into the rhombohedral, tetragonal, and orthorhombic unit cells, respectively^2^. In between the icosahedra are chains (e.g., C-B-C in B_4_C). Koroglu and Thomson^2^ suggested that the impact resistance of these materials depends on icosahedra sliding. Sliding of the icosahedra is influenced by their packing, chain formation, and the elements composing the chain. A more compact and as close as possible to a cubic ideal packing is expected to provide the best impact resistance properties, but no evidence has been presented to support this idea. Limited information is available on the assessment of the impact properties of the mentioned Al-containing phases in the Al-B-C system. To the best of author’s knowledge there are only a few articles on this topic^7–9^. Reference^7^ considers α-AlB_12_ for armor in aircraft protection, while in ref. ^8^, the authors discuss fractography details in ballistic impact experiments for body armor applications. Limited literature on the impact properties of the Al-B-C materials is partially explained by the difficult synthesis and processing of these materials as single phases and dense bulks. Some phase diagrams were reported^5^, but they are not fully resolved. The stoichiometry, crystal structure and stability domains of different B_12_-like icosahedra-containing phases, including B_4_C, need further clarifications. Another problem is the quality of the available raw materials. For example, commercial powders of B_4_C are prepared by metalothermal methods^10^ and impurity elements are often detected. These elements can stabilize the main phases or generate new ones. It is noteworthy that the Al-B-C system is sensitive to the Si^11^ or N^12^ presence. The selection of the optimum processing parameters vs. raw powders and vs. targeted phase deserves extended attention.

**Figure 1:**
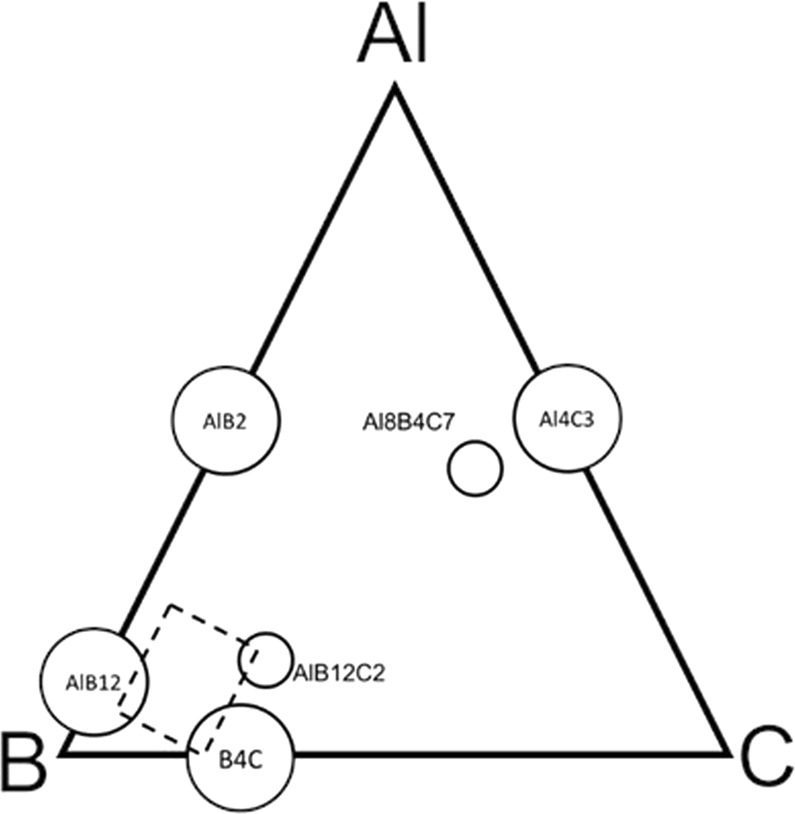
Phases in the Al-B-C system (adapted from ref. ^5^. Dashed area indicates powder mixtures investigated in the present study (see Table 1 for composition details).

Our study explores the fabrication of dense samples in the Al-B-C system by spark plasma sintering and their compressive impact resistance assessment by SHPB (Split Hopkinson Pressure Bar) tests. The sintering temperature was 1800 °C. We used as the raw powders B_4_C, α-AlB_12_ and B. The boron is crystalline and amorphous. The compositions are selected in the B-rich corner of the Al-B-C system (Fig. 1, Table 1). Structurally, the major phase is identified from the x-ray diffraction (XRD) patterns as the orthorhombic AlB_24_C_4_ when the B_4_C amount relative to α-AlB_12_ is high for the constant B-amount, i.e., when the ratio *R* = B_4_C/α-AlB_12_ ≥ 1.3. As the major phase is the most compact and close to the ideal cubic packing among the Al-B-C B_12_-type phases, the expectations according to ref. ^2^ are that samples with *R* ≥ 1.3 should have the best impact mechanical properties. Our results partially confirm this assumption; indeed, samples with *R* = 1.3 rich in AlB_24_C_4_ show the maximum Vickers Hardness (*HV*), dynamic strength (*σ*_SHPB_), strain (*e*_SHPB_), and toughness (*T*_SHPB_) values. When *R* < 1, the amount of AlB_24_C_4_ significantly decreases, new phases form and the indicated mechanical parameters rapidly deteriorate. The existence of a maximum in the curves of the mechanical parameters as a function of *R*, clearly revealed for the *σ*_SHPB_(*R*) curve, suggests that the phase assembly and the composite microstructure of the samples are also important. The use of amorphous boron promotes slightly higher values of the mechanical parameters without influencing their dependence on *R*.

**Table 1:** Samples, starting composition, density, and relative density. Indices 1 and 2 indicate amorphous (B1) and crystalline (B2) borons, respectively.

Sample	Starting Composition (wt. %)	Ratio B_4_C/α-AlB_12_ when B is normalized to 1	Theoretical density, *ρ*_T_ [g/cm^3^]	Apparent bulk density, *ρ*_a_ [g/cm^3^]	Relative density, *ρ*_R_ (%)
11	80 (80 B_4_C + 20 B1) + 20 (90 AlB_12_ + 10 B1)	3.5:1 = 3.5	2.513	2.49	99.1
12	80 (80 B_4_C + 20 B2) + 20 (90 AlB_12_ + 10 B2)	3.5:1 = 3.5	2.513	2.45	97.5
21	60 (80 B_4_C + 20 B1) + 40 (90 AlB_12_ + 10 B1)	3:2.25 = 1.33	2.520	2.47	98
22	60 (80 B_4_C + 20 B2) + 40 (90 AlB_12_ + 10 B2)	3:2.25 = 1.33	2.520	2.42	96
31	40 (80 B_4_C + 20 B1) + 60 (90 AlB_12_ + 10 B1)	2.28:3.85 = 0.59	2.527	2.49	98.5
32	40 (80 B_4_C + 20 B2) + 60 (90 AlB_12_ + 10 B2)	2.28:3.85 = 0.59	2.527	2.50	98.9
41	20 (80 B_4_C + 20 B1) + 80 (90 AlB_12_ + 10 B1)	1.33:6 = 0.22	2.534	2.46	97.1
42	20 (80 B_4_C + 20 B2) + 80 (90 AlB_12_ + 10 B2)	1.33:6 = 0.22	2.534	2.49	98.3

## Methods

### Materials and SPS processing

**Figure 2:**
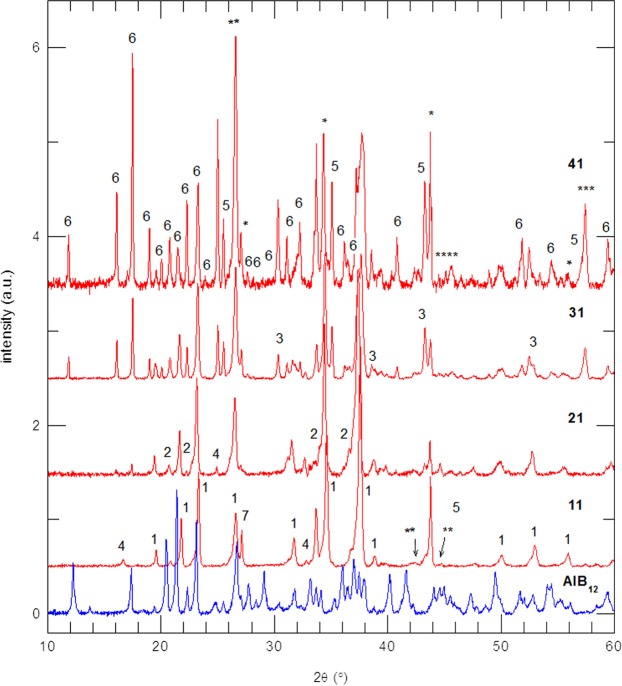
XRD patterns of the α-AlB_12_ raw powder and SPS-ed samples fabricated from amorphous boron powder (B1) from Table 1. For the sintered samples, the XRD spectra are normalized to the intensity of the peak of B_4_C at 2θ = 23.28°. Identified phases are: 1-AlB_24_C_4_ (PDF 04-008-1822), 2-Al_0.3_B_13.3_C_1.3_ (PDF 04-009-9091), 3-Al_3_BC (PDF 04-011-6299), 4-Al_4_B_2_O_9_ (PDF 00-029-0010), 5-Al_2_O_3_ (PDF 00-046-1212), 6-AlB_31_ (PDF 01-080-0621), 7- SiO_2_ (PDF 04-005-4719), *-TaB_2_ (PDF 04-003-6084), **-C (PDF 00-056-0159), ***-AlBO_3_ (PDF 00-032-0004), and ****-B_0.38_C_0.62_ (PDF – 04-014-0540).

The raw powders were B_4_C, α-AlB_12_ and B. Boron carbide was supplied by Kojundo Chemical Laboratory Co., Ltd, Japan. The BC powder based on energy dispersive spectroscopy (EDS) showed traces of impurity elements (about 1% wt.) such as Ca, Mg, Si, Fe, Cu, and Na. The powder of α-AlB_12_ was synthesized from B_4_C and Al powders in a vacuum at 1400 °C followed by chemical leaching of the impurity phases^13^. The X-ray diffraction (XRD) pattern is presented in Fig. 2. Boron was used in two forms; amorphous denoted B1 produced by Chim Reactiv co., Ltd., Donetsk, Ukraine, and crystalline denoted B2 supplied by Wako Pure Chemical Industries, Ltd., Osaka, Japan. The XRD patterns and other details of these boron powders were reported in ref. ^14^. The raw powders were mixed in ethanol using a plastic jar and balls. The starting compositions are presented in Table 1. After drying in air at 100 °C, the powder mixtures were screened through sieves of 200 and 400 mesh (74 and 37 μm).

The powder mixtures were wrapped in Ta-foil (Sigma-Aldrich Chemie, 0.025 mm thick), then in graphite foil and placed in a graphite die system. The loaded dies were placed in the processing chamber of a ‘Dr. Sinter’ SPS apparatus (Sumitomo, Japan).

Preliminary SPS experiments to find the sintering window were conducted using α-AlB_12_ powder. For the pressure of 100 MPa, the sample was heated at 50 °C/min up to 2000 °C and displacement of the punches was *in-situ* recorded by the SPS machine. By this procedure it was established that a temperature of 1800 °C is necessary for sample consolidation. At temperatures of 1400 and 1600 °C, the AlB_12_C_2_ phase forms according to refs. ^2,3^. respectively. The AlB_24_C_4_ phase was not found implying that its stability domain is at higher temperatures. Therefore, the selected SPS temperature of 1800 °C was expected to promote not only fabrication of high density bulk samples, but also reactive formation of the AlB_24_C_4_ phase.

Considering the preliminary SPS experiments, the samples from Table 1 were processed at 1800 °C for 6 min under flowing of Ar gas (2 l/min). Furnace cooling was used. The samples were 10 mm in diameter and ∼3 mm thick.

### Materials characterization

The apparent bulk density *ρ*_a_ (Table 1) of the SPS-ed samples was determined by Archimedes method using ethanol and according to ASTM B 963–08. The relative density *ρ*_R_ (Table 1) was estimated as the ratio between the apparent (*ρ*_a_) and theoretical densities (*ρ*_T_). The theoretical density was calculated considering the starting compositions and theoretical densities of B_4_C, α-AlB_12_, and B (2.54, 2.51 and 2.37 g/cm^3^).

X-ray diffraction (XRD) measurements were made using a Bruker AXS D8 Advance diffractometer (Cu_Kα_ radiation).

The microstructure and fractography details of the SPS-ed samples were observed by scanning electron microscopes (SEM, Tescan Lyra 3 and Hitachi SU 8000) equipped with energy dispersive spectroscopy (EDS) detectors. Investigations by transmission electron microscopy were undertaken by a JEM 2100 TEM.

The average Vickers hardness (ASTM C 1327–15) was determined for at least 8 indentations performed at a load of 9.8 N (1 Kgf) using a MMT-7 tester produced by Matsuzawa Seiki Co., Ltd., Japan.

The uniaxial dynamic compression tests of the SPS-ed samples from Table 1 were conducted at the high strain rates of approximately 1000 s^−1^ in the SHPB system, which has been successfully used to characterize various other ceramics including silicon carbide^15^, alumina^16,17^, boron carbide^18,19^ and MgB_2_^20^. The end surfaces of the SPS-ed samples were polished, then examined by an optical microscope prior to mechanical testing; only the samples without surface defects were tested. The sample ends were lubricated with Castrol LMX grease to minimize the interfacial friction. The SHPB system consisted of a 20-mm diameter YAG300 maraging steel striker (length 400 mm), input (length 1200 mm) and output (length 1200 mm) bars. A pair of wave impedance-matched cylindrical tungsten carbide inserts (17-mm diameter and 17-mm length) was sandwiched between the bars and specimen to prevent any indentation into the steel bars by the hard ceramic sample. The steel sleeves were used to confine and further strengthen the inserts such that they could remain intact prior to the sample failure. Both the input and output bars were instrumented with TML strain gauges (Tokyo Sokki Kenkyujo Co., Ltd., Japan, gauge factor of 2.11). Signals recorded from the strain gauges were used to calculate the stress and strain histories based on the one-dimensional elastic bar wave theory for a pulse propagating in a uniform bar. The SHPB dynamic toughness (*T*_SHPB_) was evaluated as the area below the measured strain–stress curve. The details about the SHPB system and subsequent analysis of the measured strain waves can be found in previous reports^16,17^.

## Results

### Phase assembly and microstructure of the bulk samples obtained by SPS

The XRD patterns of the SPSed samples (Table 1) fabricated from the amorphous boron raw powder B1 are presented in Fig. 2. Similar results were recorded for the samples fabricated by using the crystalline boron powder B2 (Fig. 1 Supplementary Material). The following observations are of interest:

(i) The α-AlB_12_ peaks in the XRD pattern of the raw powder cannot be visualized in the patterns of the sintered samples. This suggests that this phase is consumed during the SPS heating processes reacting with B_4_C and B to form Al boride (AlB_31_), borocarbide (AlB_24_C_4_, Al_0.3_B_13.3_C_1.3_, Al_3_BC), oxide (Al_2_O_3_) or boroxide (Al_4_B_2_O_9_) phases. Traces of AlBO_3_ and B_0.38_C_0.62_ are also possibly present in our samples, but their identification is difficult due to their low amount. Phases TaB_2_ and free-C are residuals from the surface of the sintered samples due to the Ta and C foils used in the SPS processing. Some peaks were ascribed to SiO_2_ from the XRD glass holder.

(ii) Use of a different type of boron, amorphous (B1) or crystalline (B2), does not have a significant influence on the XRD patterns (compare patterns for samples ‘11’ and ‘12’, ‘21’ and ‘22’, ‘31’ and ‘32’, ‘41’ and ‘42’ from Fig. 2 and Fig. 1 in Supplementary Material). This result also suggests a good reproducibility of the SPS processes for a fixed starting composition.

(iii) There are two groups of patterns. In the first group are samples ‘11’ (‘12’) and ‘21’ (‘22’). The major phase in the samples with a high ratio (*R*) of B_4_C/α-AlB_12_ (*R* ≥ 1.3, i.e., samples ‘11’ (‘12’) and ‘21’ (‘22’) when B is normalized to 1, is the orthorhombic Al-BC B_12_-type phase, AlB_24_C_4_. The impurity phases are Al_0.3_B_13.3_C_1.3_, Al_4_B_2_O_9_, and Al_2_O_3_. The difference between samples ‘21’ (‘22’) (*R* = 3.5) and ‘11’ (‘12’) (*R* = 1.3) is a higher amount of the Al_0.3_B_13.3_C_1.3_ and Al_2_O_3_ phases in the first samples. This suggests that when the amount of AlB_12_ is low (*R* = 3.5), Al from this phase is mainly used to obtain the solid solution between Al and B_4_C, i.e., the phase AlB_24_C_4_. At higher amounts of AlB_12_ (*R* = 1.3), Al from AlB_12_ oxidizes and also participates in the formation of new BC phases such as Al_0.3_B_13.3_C_1.3_. When the amount of AlB_12_ further increases and the ratio B_4_C/α-AlB_12_ decreases below 1 (i.e., samples ‘31’ (‘32’) and ‘41’ (‘42’)) the amount of secondary phases vs. AlB_24_C_4_ significantly increases with a shift in the equilibrium towards formation of new phases such as AlB_31_ and Al_3_BC. Considering this change in the behavior, a second group of patterns is defined for samples ‘31’ (‘32’) and ‘41’ (‘42’).

The two groups of samples identified by XRD are supported by electron microscopy observations and by mechanical properties that will be addressed in the next Section. When normalized to C, the SEM/EDS composition of the matrix in samples ‘11’ (‘12’) and ‘21’ (‘22’) from the first group is Al_0.04-0.07_B_3.3-5.6_C, while for samples ‘31’ (‘32’) and ‘41’ (‘42’) from the second group, it is Al_0.5-0.7_B_3.6-7_C. One observes that in the matrix from the samples in the second group (*R* < 1), there is about 10 fold more Al than in the first group. This is in good agreement with the XRD observation that AlB_24_C_4_ (or Al_0.25_B_6_C when normalized to C) is the major phase in the first group (*R* ≥ 1.3) and, in the second group, the amount of other phases with a higher amount of Al (e.g., Al_3_BC) is high. However, we note that the SEM/EDS compositions are often found to be different from the stoichiometry of the phases proposed by the Powder Diffraction Files (PDF) and identified in our XRD spectra (Fig. 2). This issue is often mentioned in the literature (e.g.^2,9^) and it needs further study. The reason is, on the one hand, the small dimensionality of the observed phases vs. the larger electron spot size in EDS, and, on the other hand, the fundamental uncertainties related to the structure and stoichiometry of the Al-BC phases make difficult a deep analysis of the experimental data and caution is necessary to avoid misleading conclusions.

**Figure 3:**
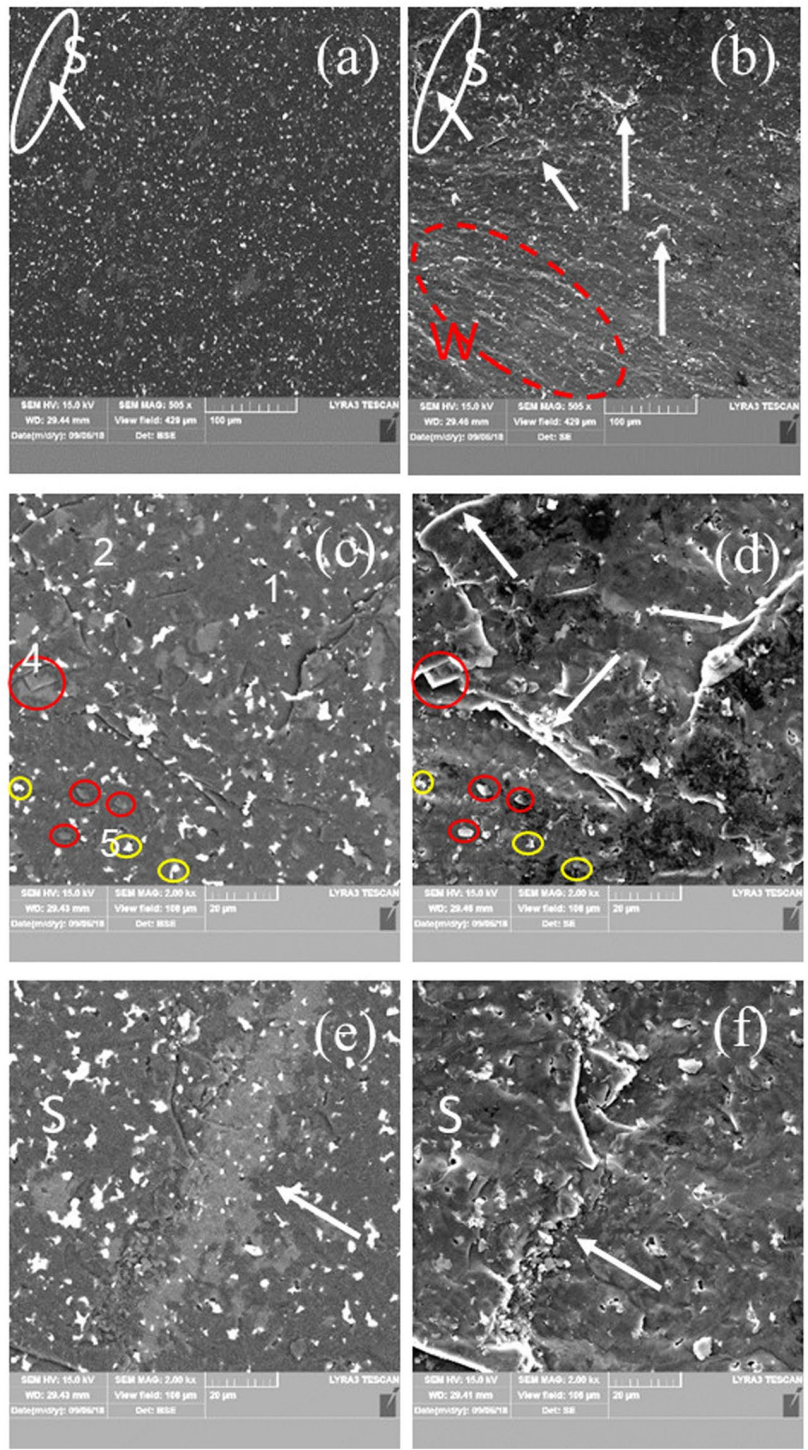
SEM micrographs of sample ‘21’ at different magnifications taken on a freshly fractured surface. Images (**a,c,e,b,d,f**) are taken in the backscattering and secondary electrons modes. Ascribed phases in BSE mode are: 1 - AlB_24_C_4_ (dark matrix), 2 - Al_0.3_B_13.3_C_1.3_ (dark gray impurity phase of relatively large size), 4 - Al_4_B_2_O_9_ (light gray impurity phase of relatively small size indicated within red circles), and 5 – Al_2_O_3_ (white small impurity grains indicated within yellow circles). ‘Steps’ are marked by arrows. Steps area S from (**a,b**) is presented at a higher magnification in (**e,f**). In (**b**), W is a region with ‘waves’.

**Figure 4:**
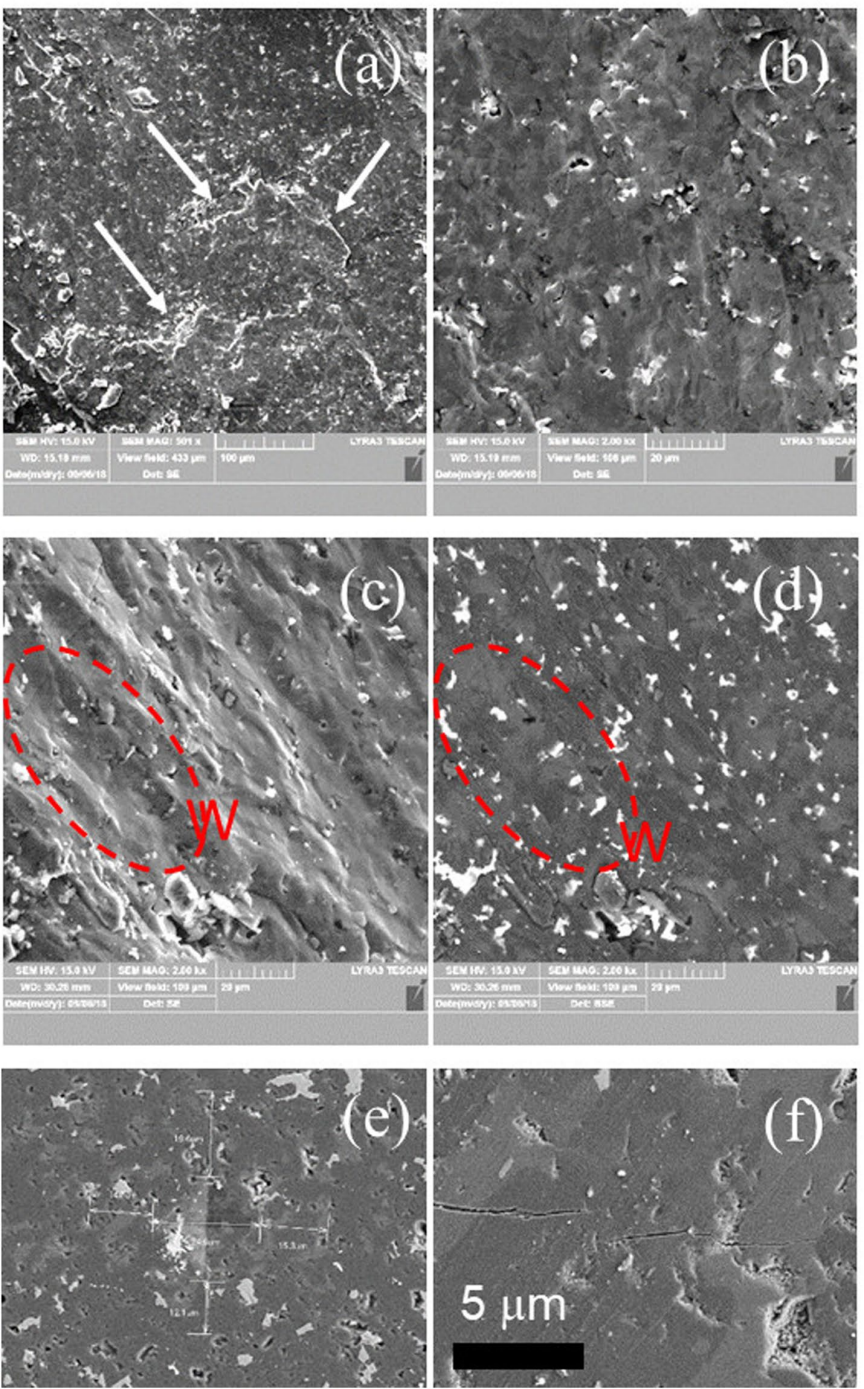
SEM micrographs of sample ‘31’ at different magnifications of: (**a**–**d**) - a freshly fractured surface (static load) and (**e,f**) - on a polished surface. Images (**a**–**c**,**d**–**f**) are taken in the secondary electrons and backscattering modes, respectively. In images (**e,f**), one can visualize the Vickers imprint and the resulting cracks. ‘Steps’ are indicated by arrows and a ‘waves’ region is marked by W.

**Figure 5:**
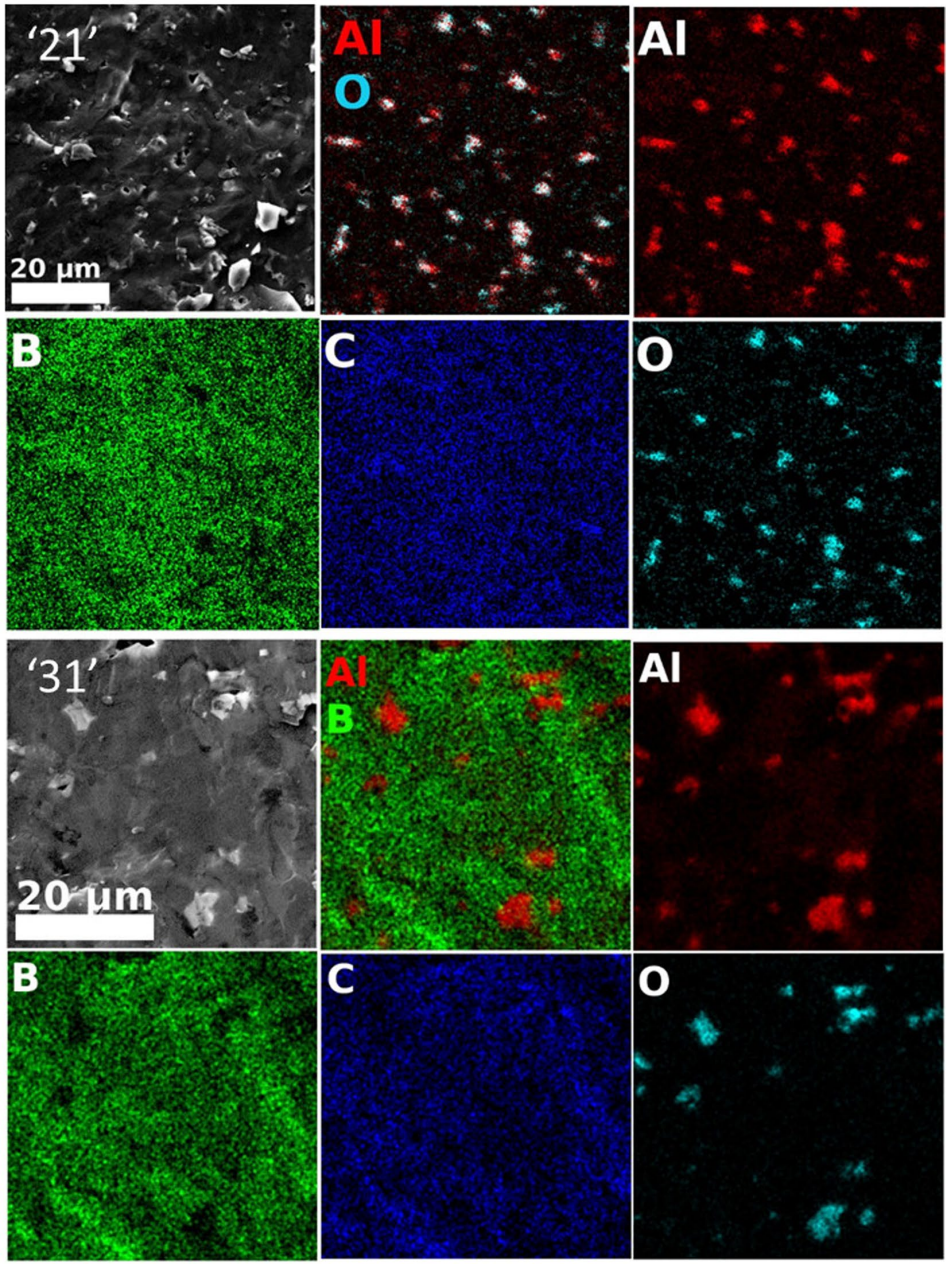
EDS elemental maps of Al, B, C and O taken of samples ‘21’ and ‘31’. Red-green-blue (RGB) images obtained by overlapping the maps of Al and O for sample ‘21’ and Al and B for sample ‘31’ are also presented.

**Figure 6:**
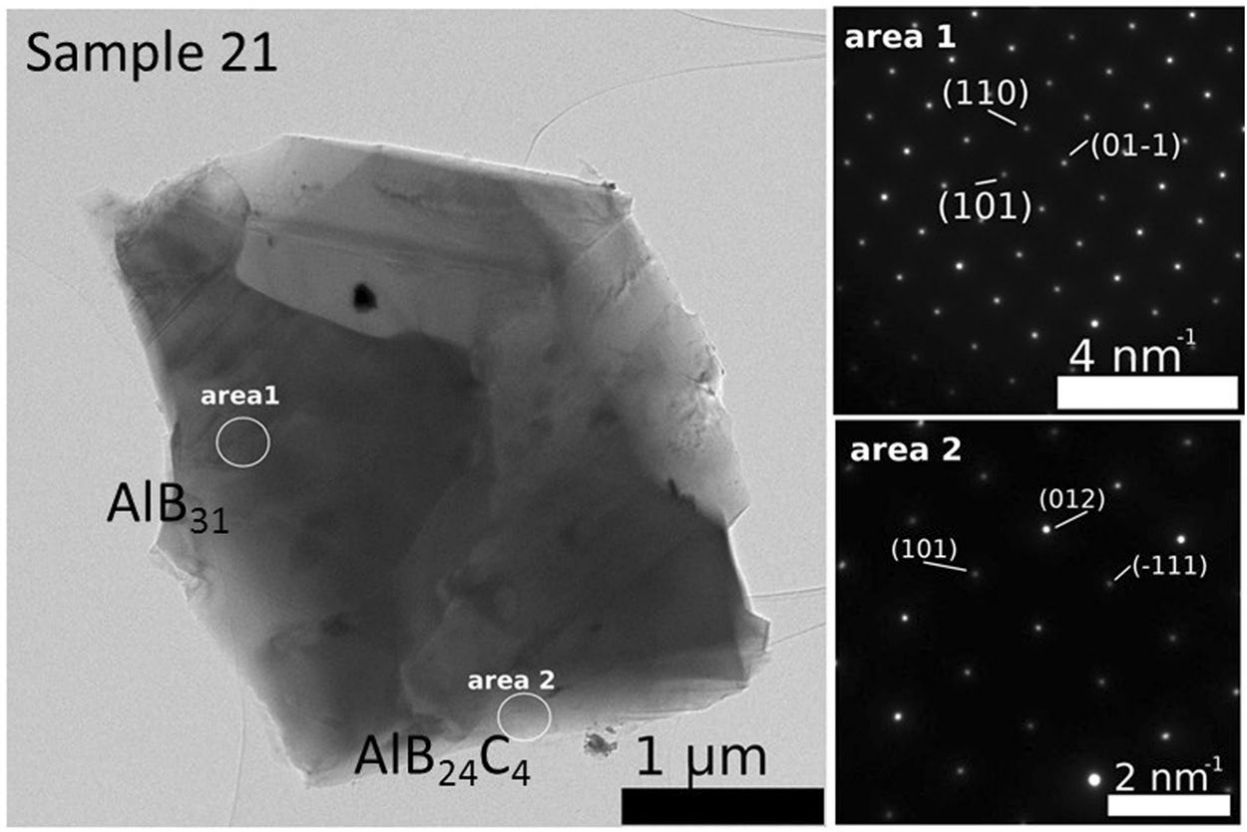
TEM and SAED micrographs taken of sample ‘21’.

The microstructures observed in the secondary (SE) or back scattering (BSE) electrons of samples ‘21’ and ‘31’ are presented in Figs. 3 and 4 while the EDS maps are in Fig. 5. The TEM results for both samples are shown in Figs. 5 and 6.

In the BSE mode, the phases can be distinguished according to their gray nuance and also considering the EDS maps (Fig. 5), they are ascribed to the phases identified in the XRD. A phase containing much of the heaviest element from the studied system, i.e., Al will have a lighter gray color in the BSE contrast image.

For sample ‘21’, the darkest black phase is associated with the matrix phase AlB_24_C_4_ (Fig. 3a,c,e) which is the major phase according to the XRD. In the matrix are embedded secondary phases with a relatively higher amount of Al; dark gray phase Al_0.3_B_13.3_C_1.3_, light gray phase Al_4_B_2_O_9_, and white phase Al_2_O_3_. According to the XRD some AlB_31_ is also possibly available in this sample, although the amount of this phase significantly enhances in the samples from the group 2 (‘31’ (‘32’) and ‘41’ (‘42’)). The grains of the dark gray phase, (Al_0.3_B_13.3_C_1.3_) are of 10–20 μm diameter and have an irregular shape. There are also extended and elongated regions of this phase of a large size (∼100 μm length, Fig. 3e). Aluminum-based oxide grains are the smallest ones, have mostly a plate- or bar- like morphology sometimes with sharp edges and tips of ∼120°, and they are present in all the sintered samples. (see Fig. 5).

For sample ‘31’, in the SEM micrographs taken in the BSE mode from Fig. 4d,f, two dark gray phases defining the matrix can be observed with difficulty. These phases show an irregular morphology of an extended size. In the matrix of sample ‘31’, as in the case of sample ‘21’, are embedded Al-based oxide phases. Their size in sample ‘31’ is larger than for sample ‘21’. According to the XRD, the major phases to form the matrix are Al_0.3_B_13.3_C_1.3_, AlB_31_ and Al_3_BC. The highest relative amount of Al is for phase Al_3_BC, and based on this result, we propose that in the BSE mode the light gray phase in the matrix is this phase, while a distinction between phases Al_0.3_B_13.3_C_1.3_ and AlB_31_ is not possible.

**Figure 7:**
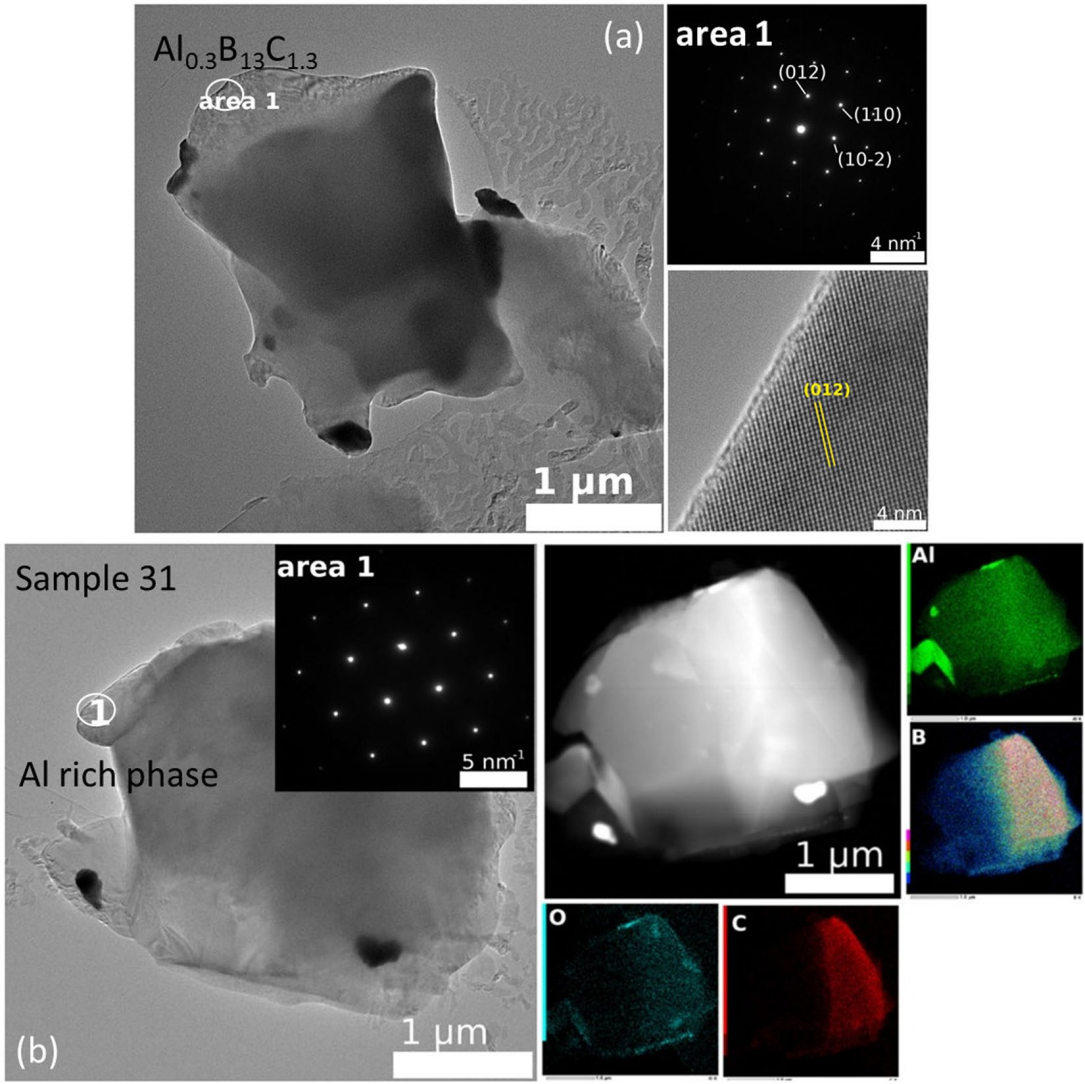
(**a**) TEM, SAED and HTEM micrographs and (**b**) TEM, SAED and EDS elemental maps of Al, B, C, and O taken of sample ‘31’.

Our analysis up to this level of presentation is based on the assumption that the stoichiometry of the phases observed by XRD is as proposed in the literature and in the powder diffraction (PDF) files. We also take into account the phase evolution as determined from variation in the XRD patterns (Fig. 2) when the starting composition is systematically modified (Table 1). Actually, the local EDS measurements in the TEM present a complex situation in which there are some unresolved details deserving attention. The selected area electron diffraction (SAED) pattern in Fig. 6, area 1 is identified with the structure of AlB_31_ (Al_0.031_B, normalized to B), but the experimental EDS composition (Al_0.023–0.047_BC_0.016–0.018_, normalized to B) shows the presence of C inside this phase (Table 2). This phase is identified by SAED in samples ‘21’ and ‘31’. Typical for sample’21’ from group 1 is the SAED pattern from Fig. 6, area 2. The stoichiometry as determined by EDS (Table 2) is Al_0.011–0.0175_BC_0.058–0.097_ and it is Al and C deficient when compared to the theoretically accepted one for the XRD majority phase AlB_24_C_4_ (written as Al_0.042_BC_0.167_ when normalized to B). For sample ‘31’ from the second group, apart from AlB_31_, another typical phase is Al_0.3_B_13_C_1.3_ (Al_0.023_BC_0.1_, normalized to B). The experimental EDS stoichiometry (Table 1) is Al_0.02_BC_0.097_ (normalized to B). The theoretical and experimental stoichiometry well matches each other, while the SAED and HTEM patterns (Fig. 7a) correspond to the phase Al_0.3_B_13_C_1.3_. Despite the apparently good theoretical and experimental agreement, it is important to note that a clear identification between AlB_24_C_4_ and Al_0.3_B_13_C_1.3_ is not possible. This is because the crystal structures are similar and the SAED patterns cannot distinguish fine structural details, while the EDS data show a high Al and Al/C ratio scattering (Table 2). The XRD evolution provides additional useful information that allows some guidance, but in some cases, unidentified phases are observed. An example of an Al-rich BC phase (Al_0.126–0.224_BC_0.094–0.11_, Table 2) in sample ‘31’ with an identified structure from SAED is presented in Fig. 7b. The phase does not contain oxygen and this can be easily observed in the EDS maps in which the Al-O phase is also visible.

**Table 2:** Most probable phases from the structural viewpoint identified by selected electron diffraction investigations (SAED), their theoretical and experimentally measured stoichiometry (normalized to B) and the theoretical and experimental Al/C ratio.

Sample	Phase	Normalized phase to B	Phase Ratio Al/C	Experimental EDS stoichiometry (Normalized to B)	Experimental EDS ratio (Al/C)	SAED representative image is presented in:
‘21’	AlB_31_	Al_0.031_B	—	Al_0.023–0.047_BC_0.016–0.018_	1.44–2.61	Fig. 6 area 1
	AlB_24_C_4_	Al_0.042_BC_0.167_	0.25	Al_0.011–0.0175_BC_0.058–0.097_	0.19–0.18	Fig. 6 area 2
‘31’	Al_0.3_B_13_C_1.3_	Al_0.023_BC_0.1_	0.23	Al_0.02_BC_0.097_	0.21	Fig. 7(a) area 1
	Al rich	—	—	Al_0.126–0.224_BC_0.094–0.11_	1.34–2.04	Fig. 7(b) area 1

SEM observations show also the presence of a low amount of closed sintering pores, often with round edges. The pores edges are brighter than the surroundings in the BSE contrast, thus suggesting the presence of a relatively high amount of heavier elements such as Al or other impurities. The size of the pores is below 4 μm.

The microstructural investigation of similar samples (‘11’ and ‘12’, ‘21’ and ‘22’, ‘31’ and ‘32’, or ‘41’ and ‘42’) when using amorphous (B1) and crystalline (B2) boron could not reveal any significant differences.

### Fractography analysis, and static and impact mechanical properties of the bulk Al-B-C samples

**Figure 8:**
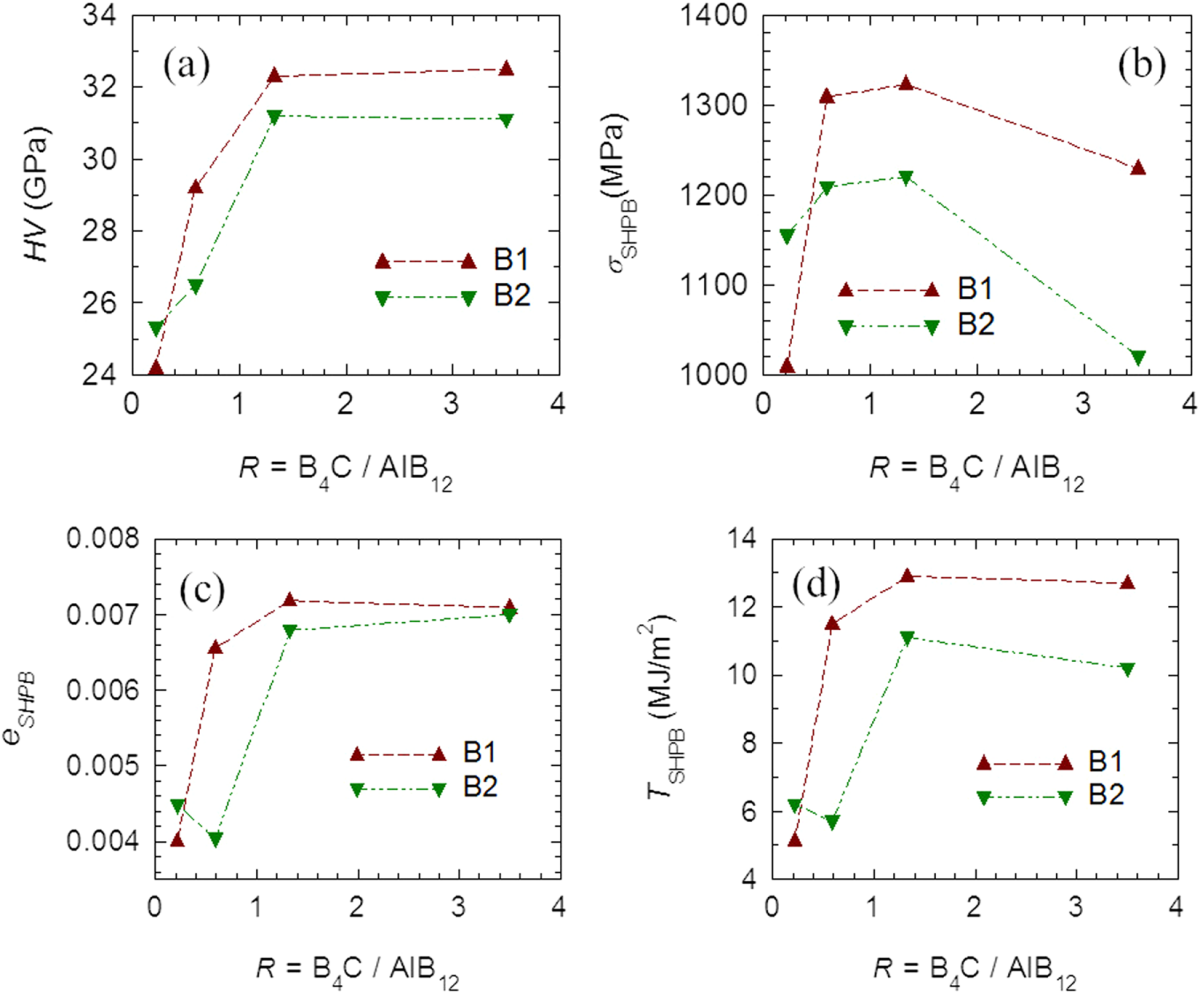
(**a**) - Vickers hardness (*HV*), (**b**) - dynamic strength (*σ*_SHPB_), (**c**) - strain (*e*_SHPB_) and (**d**) -toughness (*T*_SHPB_) as a function of the ratio (*R*) between the amount of B_4_C and AlB_12_ in the initial powder mixtures. The samples were obtained by SPS using the amorphous (B1) or crystalline (B2) boron (see Table 1).

The mechanical parameters determined for static and dynamic loading are listed in Fig. 8.

Curves of the Vickers Hardness *HV*(*R*), dynamic strain *e*_SHPB_(*R*), and dynamic toughness *T*_SHPB_(*R*) for each type of raw boron (amorphous B1 or crystalline B2) show a plateau for the AlB_24_C_4_-rich samples with *R* = 1.3–3.5 from the first group. A decrease in *R* below 1.3 results in a rapid decrease of the indicated parameters. As already addressed in the previous Section, in samples from the second group with *R* < 1.3, the amount of AlB_24_C_4_ is low and equilibrium shifts towards the formation of a significant amount of new phases such as Al_0.3_B_13.3_C_1.3_, AlB_31_ and Al_3_BC. The results indicate the strong and positive influence on the mechanical parameters of the AlB_24_C_4_. This partially confirms the prediction from ref. ^2^ of the highest impact mechanical properties for the AlB_24_C_4_ phase.

However, one observes that the curves of the dynamic strength *σ*_SHPB_(*R*) show a shape with a maximum located at *R* = 1.3. This result suggests that the presence of secondary phases and the composite microstructure of the AlB_24_C_4_-rich samples (for *R* ≥1.3) improves the dynamic strength. In the AlB_24_C_4_-poor samples, for the decreasing *R* (*R* < 1.3), *σ*_SHPB_ decreases following the similar trend of the *HV*(*R*), *e*_SHPB_(*R*), and *T*_SHPB_(*R*) curves. To reveal the strengthening mechanisms, the fractography analysis is addressed in the next paragraphs.

**Figure 9:**
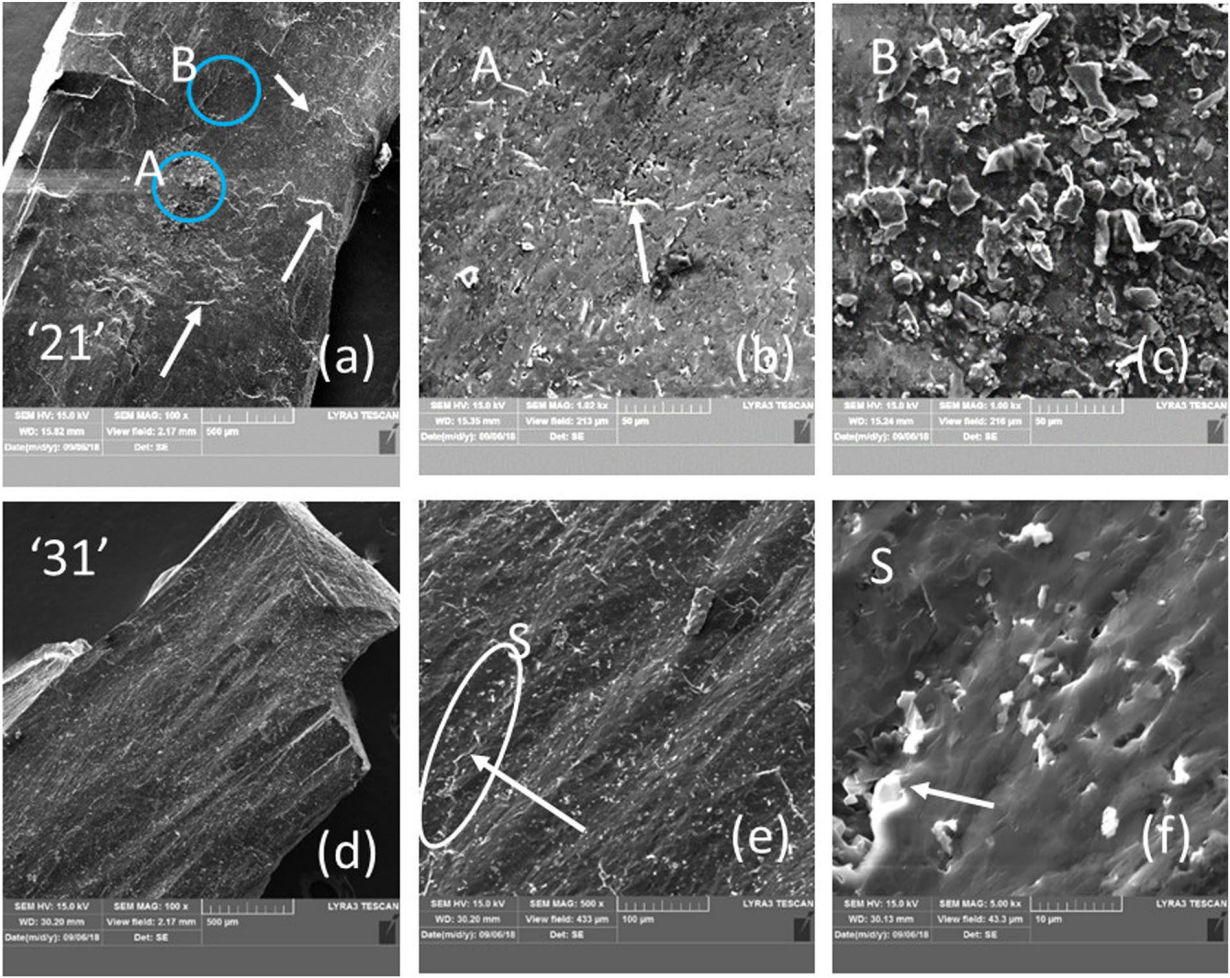
SEM micrographs of the surface of the pieces from samples ‘21’ and ‘31’ at different magnifications after the SHPB impact test. The details of regions A, B and S from (**a**,**e**) are presented in (**b**,**c**,**f**), respectively. The arrows indicate ‘steps’.

Fractured surfaces of samples ‘21’ and ‘31’ obtained under quasi static and impact loadings are presented in Figs. 3, and 9 (sample ‘21’) and in Figs. 4 and 9 (sample ‘31’), respectively. The surfaces are typical for the brittle fracture by a transgranular mechanism. For example, the crack in Fig. 4f linearly develops under quasi static loading in the HV indentation over different large-size phases. Nevertheless, from the same image, it is also visible that the crack’s bridging and deflection occur when small impurity phases interfere with the crack. This effect and an inter-granular sliding with a ‘pull out’ of the small Al-based oxides (Fig. 3c,d) provides ductility to our samples. Some plasticity is also inferred from the wavy fractured surface (denoted W) resulting from both static (Figs. 3b, 4c) and dynamic loadings (Fig. 9e,f). Other elements, as evidence for the plasticity, are the ‘steps’ (Figs. 3b,d,f, 4a and 9a,b,e,f, follow the arrows and regions S). The formation of the wavy surface and the ‘steps’ is related to the presence of large and softer phases than AlB_24_C_4_ (compare Fig. 3e with f, Fig. 4c,d, and see Fig. 9e,f region S). Depending on the type, amount, size, morphology and distribution of these phases, the pattern of the fractured surface is modified and, thus, it serves as a fingerprint of the changing mechanical parameters. We note that in the dynamically fractured surface of sample ‘21’ are visible less ‘waves’ perhaps due to the lower concentration of the secondary large and soft phases relative to the amount of the hard AlB_24_C_4_ than in sample ‘31’. On the other hand, in sample ‘21’ apart from the flat surfaces (region A in Fig. 9a,b), regions with small fractured grains may occur (region B, Fig. 9a,c). Large regions of a secondary phase (ascribed mainly to Al_0.3_B_13.3_C_1.3_) composed of small grains resulting from fracturing and which defines a ‘step’ or more are also observed in Fig. 3e,f for sample ‘21’ fractured under quasi static loading. Fracturing of the large regions of the secondary phases into small grains and considering the irregular morphology of these phases and of their irregular interface with the Al-BC main phase may indicate a mechanical anchoring of the phases. It is inferred that these kinds of ‘reinforced’ grain boundaries in the composite can provide for the optimum phase assembly and microstructure an enhancement of the dynamic strength as observed for our samples with *R* = 1.3. This result deserves attention as a useful and general route to control and improve the dynamic properties of hard ceramic composite materials, but further research is necessary.

The described dependencies are preserved when using different boron types, but use of an amorphous boron increases the values of Vickers Hardness (*HV*), dynamic strength (*σ*_SHPB_), strain (*e*_SHPB_), and dynamic toughness *T*_SHPB;_ the maximum values in the sample with *R* = 1.3 are 32.4 GPa, 1323 MPa, 0.0072, and 12.9 MJ/m^2^, respectively. The reason why amorphous boron leads to better mechanical properties is unclear. We believe that this is related to the different reactivity of the two types of boron (expected higher for the amorphous form). In our previous study of the B_4_C samples, the values of the dynamic strength measured by the SHPB machine used in this article attained maximum values of 1400 MPa and 1270 MPa; in the first case, the sample was obtained by SPS in a vacuum at 1600 °C under a high uniaxial pressure of 300 MPa, and in the second by SPS in nitrogen at 1800 °C under the uniaxial pressure of 100 MPa^18,19^. The maximum value of *σ*_SHPB_ determined for the Al-B-C composite from this study is relatively high, and comparable to our best values for B_4_C, thus enabling the use of this material in different applications.

## Conclusion

High-density samples of Al-B-C were prepared by reactive spark plasma sintering and were characterized by compressive impact tests by the Split Hopkinson Pressure Bar method. The raw materials were the B_4_C, α-AlB_12_ and B powders. Boron was used in the amorphous or crystalline forms. When the ratio *R* = B_4_C/α-AlB_12_ ≥ 1.3, the main phase in the samples is AlB_24_C_4_, and when *R* < 1 other phases occur and the amount of AlB_24_C_4_ significantly decreases. The highest Vickers hardness, dynamic strength, strain and toughness are obtained for samples with *R = *1.3. The orthorhombic phase, AlB_24_C_4_, is the most compact with the closest packing to the ideal cubic one among the Al-B-C phases containing B_12_-type icosahedra. As a consequence of this feature, the literature predicts the highest impact properties for the AlB_24_C_4_ among all the Al borocarbide phases. Our results partially support this assumption, but the presence of other phases and specifics of the microstructure also play an important role. Although the type of boron does not influence the observed material and the mechanical properties dependences, slightly higher values of the dynamic mechanical characteristics are determined for samples fabricated with the amorphous boron.

## Supplementary information

Supplementary information
