# Supplementary material for: Bulks of Al-B-C obtained by reactively spark plasma sintering and impact properties by Split Hopkinson Pressure Bar
Source: Sci Rep. 2019 Dec 20;9:19484. doi: 10.1038/s41598-019-55888-z (PMC6925236; doi:10.1038/s41598-019-55888-z)
Supplement: Supplementary file 1 — Supplementary information [file 41598_2019_55888_MOESM1_ESM.docx]

**Bulks of Al-B-C obtained by reactively spark plasma sintering and impact properties by Split Hopkinson Pressure Bar**

O. Vasylkiv^1*^, H. Borodianska^1^, D. Demirskyi^1,2*^, P. Li^3,4^, T.S. Suzuki^1^, M. Grigoroscuta^5^, I. Pasuk^5^, A. Kuncser^5^, P. Badica^5*^

**Fig. 1 Supplementary material** XRD patterns of SPS-ed samples fabricated from crystalline boron powder (B2) from Table 1. For the sintered samples, the XRD spectra are normalized to the intensity of the peak of B_4_C at 2θ=23.28°. Identified phases are: 1-AlB_24_C_4_ (PDF 04-008-1822), 2-Al_0.3_B_13.3_C_1.3_ (PDF 04-009-9091), 3-Al_3_BC (PDF 04-011-6299), 4-Al_4_B_2_O_9_ (PDF 00-029-0010), 5-Al_2_O_3_ (PDF 00-046-1212), 6-AlB_31_ (PDF 01-080-0621), 7- SiO_2_ (PDF 04-005-4719), *-TaB_2_ (PDF 04-003-6084), **-C (PDF 00-056-0159), ***-AlBO_3_ (PDF 00-032-0004), and ****-B_0.38_C_0.62_ (PDF – 04-014-0540).
